# Sustained Trichodysplasia Spinulosa Polyomavirus Viremia Illustrating a Primary Disseminated Infection in a Kidney Transplant Recipient

**DOI:** 10.3390/microorganisms9112298

**Published:** 2021-11-05

**Authors:** Marie-Céline Zanella, Damien Pastor, Mariet C. W. Feltkamp, Karine Hadaya, Samuel Cordey, Laurence Toutous Trellu

**Affiliations:** 1 Division of Infectious Diseases, Geneva University Hospitals, 1205 Geneva, Switzerland; Samuel.Cordey@hcuge.ch; 2 Laboratory of Virology, Division of Infectious Diseases and Division of Laboratory Medicine, Geneva University Hospitals, 1205 Geneva, Switzerland; 3 Department of Dermatology, Geneva University Hospitals, 1205 Geneva, Switzerland; Damien.Pastor@hcuge.ch (D.P.); Laurence.Trellu@hcuge.ch (L.T.T.); 4 Department of Medical Microbiology, Leiden University Medical Center, 2333 ZA Leiden, The Netherlands; m.c.w.feltkamp@lumc.nl; 5 Department of Nephrology and Hypertension, Geneva University Hospitals, 1205 Geneva, Switzerland; Karine.Hadaya@hirslanden.ch; 6 Faculty of Medicine, University of Geneva, 1206 Geneva, Switzerland

**Keywords:** trichodysplasia spinulosa polyomavirus, polyomavirus, trichodysplasia spinulosa, transplantation, kidney, virus

## Abstract

Novel human polyomaviruses (HPyV) have been recently identified in solid organ transplant recipients. Trichodysplasia spinulosa (TS) is a rare disease associated with immunosuppression and induced by a polyomavirus (TSPyV). We report here a case of primary and disseminated TSPyV infection after kidney transplantation with extensive skin lesions, sustained viremia, and high viral loads in urine specimens, anal, nasal and throat swabs, assessed via specific real-time PCR for TSPyV during a follow-up period of 32 months after transplantation. The detection of TSPyV with a high viral load in respiratory and anal swab samples is compatible with viral replication and thus may suggest potential respiratory and oro-fecal routes of transmission.

## 1. Introduction

Beyond BK and JC polyomaviruses, novel human polyomaviruses (HPyV) that have been recently identified should be on the radar of clinicians involved in the clinical management of solid organ transplant recipients [1]. Specifically, trichodysplasia spinulosa (TS) is a rare skin disease, affecting mostly solid organ transplant (SOT) recipients, that was first reported by Izakovic et al. in 1995 [2]. TS is associated with trichodysplasia spinulosa polyomavirus (TSPyV), a human polyomavirus that was first identified in 2010 in cutaneous spicules of a heart transplant patient presenting with typical lesions of TS [3]. TS still represents a diagnostic challenge, as molecular and/or serological routine assays are not implemented in the vast majority of microbiology laboratories. We report here a case of primary and disseminated TSPyV infection during a follow-up period of 32 months after transplantation.

## 2. Materials and Methods

### 2.1. Detection and Quantification of Viral DNA

Skin biopsy specimens were disrupted in 800 μL of viral transport medium using the TissueRuptor (QIAGEN, Valencia, CA, USA) homogenizer. The viral genome was extracted individually from 190 μL of serum, plasma, native urine, homogenized skin biopsies, and nasal, throat or anal swabs (ESwabs 1 mL; Copan, Brescia, Italy), spiked with 10 μL of standardized canine distemper virus (CDV) as an internal control, using the NucliSENS easyMAG (bioMérieux, Geneva, Switzerland) nucleic acid kit and eluted in 25 μL. The TSPyV real-time PCR analysis was performed from 5 μL of eluate using the TaqMan^®^ Universal PCR Master Mix with the primers (TSPyVa-F and TSPyVa-R) and probe published by Urbano P.R. and colleagues [4] under the same cycling conditions on a QuantStudio^®^ 5 instrument (Applied Biosystems, Rotkreuz, Switzerland).

For the serum, plasma, native urine and swabs samples, the TSPyV genome concentration (upper and lower limits of quantification = 1.3 × 10^8^ and 1.3 × 10^3^ viral copies/mL, respectively; slope = 3.307; R2 = 0.998) was estimated using a standard curve previously obtained by 10-fold serial dilutions of a plasmid containing the real-time PCR target region. The CDV real-time RT-PCR assay was performed as previously described [5]. For the skin biopsy specimens, the AGPAT housekeeping gene was tested using real-time PCR to evaluate the whole process efficiency, with the primers and probe published by Merla G. and colleagues [6].

### 2.2. TSPyV IgG Seroresponse Measurement

For the measurement of IgG antibodies against TSPyV, a Luminex-based multiplex antibody binding assay was performed, as previously described [7]. In short, serum and plasma samples were mixed in a 1:100 dilution with TSPyV VP1-coupled colored fluorescent beads for 1 h at room temperature. For the detection of TSPyV VP1-bound antibodies, biotinylated goat anti-human IgG (H + L) (1:1000, Jackson ImmunoResearch Laboratories Inc.) was used, followed by streptavidine-R-phycoerythrin (1:1000, Invitrogen), each with an incubation time of 30 min at room temperature. Finally, the bead color was analyzed and the phycoerythrin signal scored in a Bio-Plex analyzer (Bio-Rad Laboratories, Hercules, CA, USA) and expressed as median fluorescent intensity (MFI). To correct for background seroreactivity, background MFI values were subtracted from the TSPyV VP1-specific signals. On every test plate, a positive serum pool was included as a control.

## 3. Results

### 3.1. Case Report

**Figure 1:**
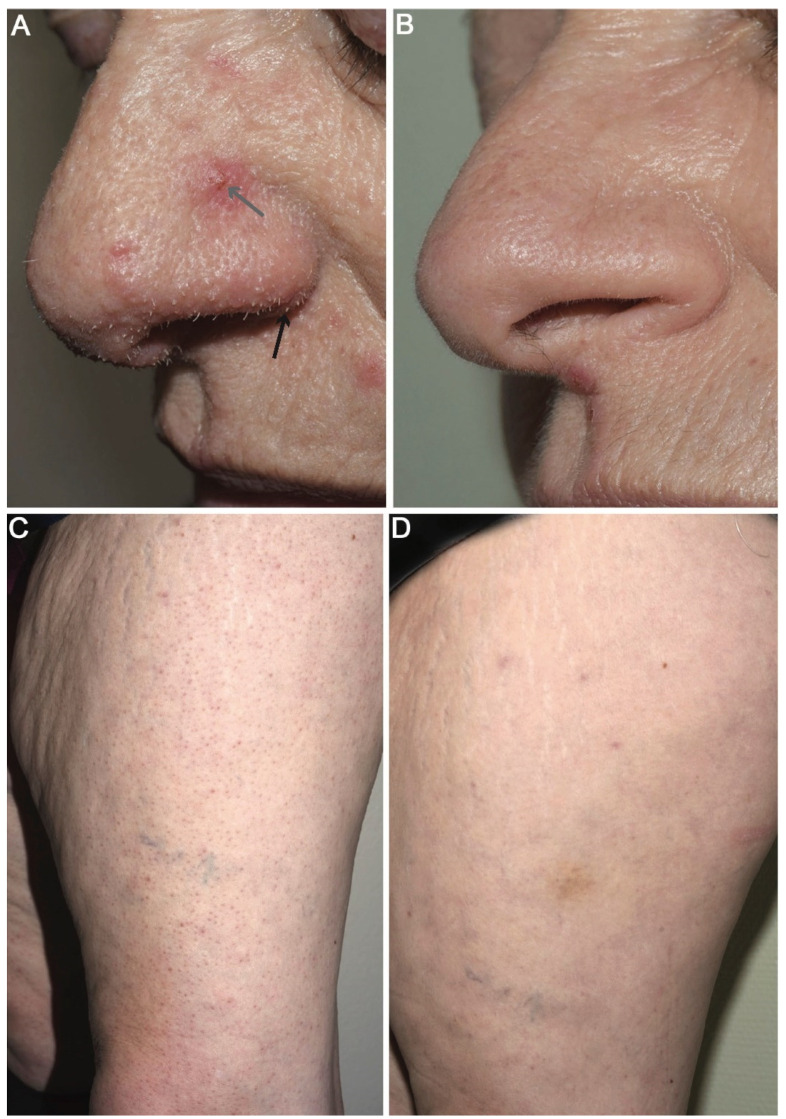
Trichodysplasia spinulosa skin lesions.

A 57-year-old female, known for IgA nephropathy, received a kidney transplant from a deceased donor on 31st October 2017. In August 2018, the patient developed skin eruptions of non-pruritic, follicular flesh-coloured papules and keratotic white spicules that started on the face and spread to the ears, back and extremities within 3 months, in addition to eyebrow alopecia. These extensive lesions were observed during a dermatological visit (February 2019) and were highly compatible with TS (Figure 1). At the time of clinical diagnosis, the patient received mycophenolate mofetil (MMF), tacrolimus and prednisone; creatinine was normal (0.94 mg/dL), and BK virus viremia was undetectable. Skin biopsies from the nose and shoulder confirmed the histopathological diagnosis of TSPyV [2,3]. We performed a specific real-time PCR for TSPyV on skin biopsy specimens collected 15 months post transplantation from typical lesions, that gave positive results (Table 1).

### 3.2. TSPyV Monitoring in Blood, Urine Samples, Anal, Nasal and Throat Swabs

**Figure 2:**
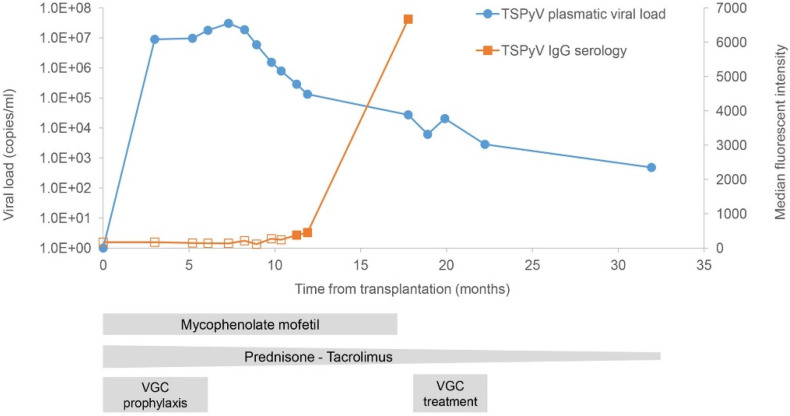
Trichodysplasia spinulosa polyomavirus (TSPyV) real-time PCR results in plasma samples and IgG seroreactivity over time after kidney transplantation. Empty and plain squares represent IgG seronegativity and seropositivity, respectively. VGC: valganciclovir.

We screened serum and plasma samples collected retrospectively from transplantation to TS onset and prospectively during follow-up visits (total period of 32 months). We also screened kidney biopsy specimens collected at the time of transplantation and at one year post transplantation as part of routine follow-up (notably, there were no histopathological signs of acute or chronic rejection in the latter). The analyses revealed a plasmatic viral load of 8.95 × 10^6^ copies/mL at 3 months post transplantation (6 months before TS onset) with a peak at 3.04 × 10^7^ copies/mL at 7 months post transplantation (2 months before TS onset) and a sustained viremia during the 32 months of follow-up (Figure 2). Other clinical samples (urine specimens, anal, nasal and throat swabs) were systematically collected during follow-up, and screening for TSPyV was positive with high viral loads in all samples during the follow-up (Table 1). In nasal swabs, despite the technical limitations of swab sampling, we observed viral loads consistently above the upper limit of quantification (LOQ) of the assay (>1.3 × 10^8^ copies/mL) during the first 22 months of follow-up, and 2.79 × 10^7^ copies/mL at 32 months. High viral loads were also systematically detected in anal swabs with 2/5 samples above the upper LOQ (Table 1). In addition, routine screening with specific PCR in plasma samples for BK polyomavirus was negative during the whole follow-up period; CMV screening was negative except at 10 and 11 months post transplantation with a low viral load (3.98 × 10^1^ and 6.45 × 10^1^ copies/mL, respectively). Interestingly, biopsies of the donor’s kidney collected during kidney transplantation and at 11.9 months post transplantation were negative and positive (Ct value = 20.901, Table 1), respectively, for TSPyV using real-time PCR (Table 1). TSPyV IgG serology was negative at the time of transplantation and positive from 11 months post transplantation (Figure 2). Furthermore, TSPyV real-time PCR and IgG serology were performed on the donor serum sample, and only IgG serology gave positive results (data not shown).

Regarding clinical management, the patient was on CMV prophylaxis with valganciclovir (450 mg od) for 6 months after transplantation (Figure 2). In February 2019, at the time of TS diagnosis (15 months post transplantation), tacrolimus and prednisone had already been tapered in a stepwise manner (from 12 to 5 mg/day and from 20 to 3 mg/day, respectively) according to kidney transplantation clinical management. At 17 months post transplantation (8 months after TS onset), immunosuppression was further reduced with discontinuation of MMF. Considering the persistence of TS lesions at 19 months post transplantation, the patient was treated for 3 months with valganciclovir (900 mg bid). Topical cidofovir is currently not commercially available in Switzerland and specific formulations need to be prepared. Regarding the disseminated lesions and considering the risk of systemic absorption of cidofovir that could have potentially been associated with renal toxicity, we would have considered using this treatment if the lesions had not improved. An almost complete regression of the lesions was observed at 22 months post transplantation. The patient’s face and upper body were clear of TS lesions, which persisted only sparsely on the lower legs and forearms (Figure 1). At 32 months post transplantation, the patient’s condition continued to improve with only rare and sparse TS lesions localized only on the knees and forearms.

Trichodysplasia spinulosa (TS) lesions with follicular micropapules (grey arrow) and keratotic spicules (black arrow) over the nose and the left leg 3 months after TS diagnosis (Figure 1A,C) and 16 months after TS diagnosis (Figure 1B,D).

## 4. Discussion

We report here a case of primary and disseminated TSPyV infection after kidney transplantation with extensive skin lesions, sustained viremia, and high viral loads in urine specimens, anal, nasal and throat swabs, during a follow-up period of 32 months after transplantation. The pre-transplantation TSPyV seronegativity of the recipient, and the negative PCR in the kidney biopsy at the time of transplantation suggest post-transplantation infection, probably not associated with the graft itself but via another and unknown route of transmission.

To date, 31 cases of TS have been reported in SOT recipients [8]. TSPyV has been detected via PCR in skin specimens [9,10,11,12,13] from transplant patients, and replicative virions have been identified with electronic microscopy and immunohistochemistry [9]. The TSPyV genome has also been detected in skin specimens [11] and tonsillar tissues of healthy individuals [14], in skin specimens [3], stool samples and nasal swabs of asymptomatic transplant recipients [15] and in urine samples of a kidney transplant recipient with TS [16], as well as in a kidney allograft biopsy specimen of a kidney transplant recipient [17]. Our observation of viremia preceding the development of skin lesions by a few months confirmed previous reports [13,16,17,18]. To the best of our knowledge, this case is the first report of sustained TSPyV viremia (over a 2-year period) [19]. The kinetics of the viremia and the IgG seroconversion 6 months after the viral load peak suggest that the reduction of the immunosuppressive regimen, the potential antiviral activity of valganciclovir and the induction of TSPyV immunity could have contributed to the clearing of the viremia. Furthermore, this study shows sustained and high viral loads in various clinical samples after transplantation, illustrating the typical protracted course of some disseminated viral infections in transplant recipients.

The mode(s) of TSPyV transmission and site(s) of latency remain undetermined to date. The detection of TSPyV in tonsillar tissue has raised questions about latency sites and respiratory/oral transmission routes [14]. Here, the identification of TSPyV with high viral load in respiratory and anal swab samples is compatible with viral replication and thus may suggest potential respiratory and oro-fecal routes of transmission. The determination of the TSPyV serostatus before immunosuppression to identify immunosuppressed patients at risk of developing TS deserves further study.

## Figures and Tables

**Table 1:** Trichodysplasia spinulosa polyomavirus (TSPyV) real-time PCR results in diverse clinical specimens of a kidney transplant recipient during a 32-month follow-up period.

Time from Transplantation	Biopsy	Urine	Nasal Swab	Throat Swab	Anal Swab
	(Ct Value)	(Copies/mL)	(Copies/mL of VTM)	(Copies/mL of VTM)	(Copies/mL of VTM)
Day 0	kidney: undetected	-	-	-	-
11.9 months	kidney: 20.90	-	-	-	-
15.4 months	skin: 13.95	-	-	-	-
15.7 months	skin: 6.54	-	-	-	-
17.8 months	-	1.82 × 10^7^	>1.3 × 10^8^	2.15 × 10^7^	>1.3 × 10^8^
18.9 months	-	1.97 × 10^7^	>1.3 × 10^8^	1.42 × 10^6^	>1.3 × 10^8^
19.9 months	-	2.84 × 10^5^	>1.3 × 10^8^	7.64 × 10^6^	3.28 × 10^7^
22.2. months	-	3.22 × 10^4^	>1.3 × 10^8^	7.96 × 10^6^	6.62 × 10^6^
31.9 months	-	1.30 × 10^4^	2.79 × 10^7^	1.09 × 10^4^	3.18 × 10^5^

Abbreviations: Ct: cycle threshold; VTM: viral transport medium in which sample swab was collected.

## Data Availability

Available on request.
